# Unanticipated Difficult Airway in a Child With an Undiagnosed Subglottic Laryngeal Web: Caught in the Web, Saved by a Nick

**DOI:** 10.7759/cureus.82729

**Published:** 2025-04-21

**Authors:** Vinitha Narayan, Swati Patel, Ashwini Reddy, Rajeev Chauhan

**Affiliations:** 1 Anesthesiology, Postgraduate Institute of Medical Education and Research (PGIMER), Chandigarh, IND; 2 Anesthesia and Critical Care, Mata Kaushalya Hospital (MKH) Hospital, Patiala, IND; 3 Anesthesia and Critical Care, Postgraduate Institute of Medical Education and Research (PGIMER), Chandigarh, IND

**Keywords:** difficult airway management, difficult pediatric airway, fibreoptic bronchoscopy, laryngeal web, otolaryngology

## Abstract

Laryngeal webs are abnormal tissue formations within the larynx, typically caused by incomplete recanalization during embryonic development of the larynx at 8-10 weeks of gestation. They result from incomplete resorption of the epithelial layer that normally obliterates the developing laryngeal opening, leading to the formation of a web. Congenital laryngeal webs are rare, with an estimated incidence of approximately 1 in 10,000 births, accounting for about 5% of all congenital laryngeal anomalies. They are associated with significant anesthetic challenges, such as difficulty in intubation or the need for invasive airway access. In pediatric patients, this is further complicated by a smaller airway diameter, lower oxygen reserves, and faster desaturation, which can quickly turn an unanticipated airway difficulty into a critical situation. Here, we present the airway management of a child with unanticipated difficult intubation secondary to an undiagnosed subglottic web.

## Introduction

Laryngeal webs are abnormal tissue formations within the larynx, typically caused by incomplete recanalization during embryonic development of the larynx at 8-10 weeks of gestation. This results from incomplete resorption of the epithelial layer that normally obliterates the developing laryngeal opening, leading to the formation of a web. Congenital laryngeal webs are rare conditions, with an estimated incidence of approximately 1 in 10,000 births, accounting for about 5% of all congenital laryngeal anomalies [1]. Laryngeal webs are uncommon congenital lesions that typically present around the age of 3 years with vocal or respiratory symptoms [2]. These webs can form in the anterior glottic area, supraglottic, or subglottic regions. Laryngeal webs may present as isolated lesions or may be associated with other congenital syndromes such as DiGeorge syndrome, 22q11.2 deletion, CHARGE syndrome, etc. [3]. They are associated with significant anaesthetic challenges, such as difficulty in intubation or the need for invasive airway access. In pediatric patients, this is further complicated by a smaller airway diameter, lower oxygen reserves, and faster desaturation, which can quickly turn an unanticipated airway difficulty into a critical situation. While only a few cases of asymptomatic patients or mild hoarseness of voice have been reported, many cases are associated with significant challenges, such as abandonment of the procedure due to intubation difficulties or the requirement for invasive airway access [4]. Here, we present the airway management of a child with unanticipated difficult intubation secondary to an undiagnosed subglottic web.

## Case presentation

**Figure 1:**
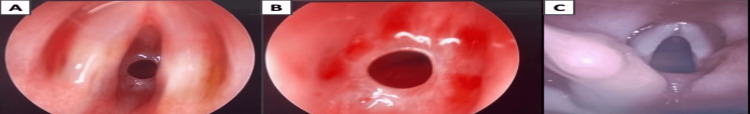
A flexible fiberoptic bronchoscope image showing a thin membranous structure (1A) with a central lumen (1B) suggestive of a laryngeal web in the subglottic region, along with an image of normal vocal cords and glottic aperture (1C).

A three-year-old child weighing 15 kg, with a pediatric Glasgow Coma Scale of E3V4M5 secondary to congenital hydrocephalus, was posted for elective endoscopic third ventriculostomy. He had no history of stridor, respiratory difficulties, or hoarseness of voice. Examination of the airway revealed adequate mouth opening, normal sternomental distance, and absence of any external deformities. The child had a head circumference of 57 cm with dilated veins over the scalp, and a chest circumference of 50 cm. After confirming adequate fasting, the child was wheeled into the operating theatre and standard monitoring was instituted. Induction was done with Injection Fentanyl 2 mcg/kg (30 mcg) and Injection Propofol 2 mg/kg (35 mg). After confirming adequate bag-mask ventilation, Atracurium 0.5 mg/kg (7.5 mg) was administered. On direct laryngoscopy, a Cormack-Lehane grade 2 view was obtained, and intubation was attempted. However, a 4.0 mm ID cuffed endotracheal tube could not be negotiated beyond the level of the vocal cords. Repeat attempts with 3.5 mm and 3.0 mm uncuffed endotracheal tubes were also unsuccessful. Further intubation attempts were then abandoned, and an I-gel™ supraglottic airway device (SGAD), size 1.5, was inserted to maintain ventilation. A pediatric flexible fiberoptic bronchoscope with a 4.9 mm internal diameter was then inserted through the SGAD to assess the subglottic region. This revealed a thin membranous structure with a small central lumen suggestive of a laryngeal web (Figures 1A-1C show normal vocal cord anatomy). An otolaryngologist was called, who passed a rigid fiberoptic bronchoscope and excised the laryngeal web, following which the trachea was successfully intubated with a 4.0 mm internal diameter cuffed endotracheal tube. Dexamethasone (0.5 mg/kg) was administered, considering the repeated intubation attempts and airway manipulation. The otolaryngologist confirmed that the child had a Type 2 Cohen’s grade laryngeal web. The neurosurgical procedure was uneventful, and extubation was performed once the child was fully awake, after the reversal of residual neuromuscular blockade. The rest of the hospital stay was uneventful, and the child was subsequently discharged after two days.

## Discussion

Laryngeal webs are rare airway anomalies that can result from both congenital and acquired causes. Congenital laryngeal webs arise from incomplete recanalization of the tracheal lumen during the 10th week of embryogenesis, typically occurring 2-3 mm below the level of the vocal cords. Depending on the degree of luminal obstruction, the child may present with stridor, hoarseness of voice, or recurrent upper respiratory tract infections [5]. In this case, an undiagnosed subglottic laryngeal web presented as an unanticipated difficult airway during the induction of anaesthesia for an endoscopic third ventriculostomy.

Several congenital anomalies have been found in association with laryngeal webs, including other airway lesions, congenital heart disease, and respiratory papillomatosis. However, the association of hydrocephalus with laryngeal webs has not been reported in the literature. The clinical features and management of laryngeal webs depend on their location and grading. Grade 1 webs are thin, membranous, and involve less than 35% of the glottic aperture; Grade 2 involves 35-50% of the glottis; Grade 3 covers 50-75% of the glottis and may extend into the anterior cricoid cartilage; while Grade 4 involves up to 99% of the glottis. We encountered a Grade 2 web, which was dissected with a knife. Simple webs are easily treated with either laser lysis or microdissection, whereas larger webs may require more complex procedures such as laryngeal reconstruction and stenting [6].

Children with subglottic webs can be asymptomatic or have mild symptoms, especially if the web is thin, thereby remaining undiagnosed until they present with unexpected difficult intubation for unrelated surgeries. An unanticipated difficult airway is particularly challenging in pediatric patients due to physiological differences, such as lower cardiopulmonary reserve and higher oxygen consumption, which reduce the safe apnoea time. Depending on the degree of luminal narrowing, a smaller-sized endotracheal tube can be inserted, or a supraglottic device can be employed for ventilation. The supraglottic device can also be used as a conduit to perform fiberoptic bronchoscopy and facilitate intubation [7]. In this case, fiberoptic bronchoscopy helped in the diagnosis of the laryngeal web, underscoring its value in the evaluation of unexplained difficult airways. Other devices used to maintain oxygenation include a nasal cannula, an endotracheal tube placed in the pharynx, a nasal trumpet connected to the circuit, and a tracheostomy [8]. This case highlights the importance of having a difficult airway cart with the availability of alternative airway devices and techniques.

A narrow margin of safety also makes it imperative to call for help early and minimize the number of intubation attempts. Our patient was already suffering from hydrocephalus, and even short periods of hypercapnia or relative hypoxia could prove disastrous. It is also crucial not to forcefully advance the tube upon encountering subglottic resistance. Nguyen NK also reported a case of a subglottic laryngeal web in an adult, in which a supraglottic device was inserted following the inability to intubate; the web was subsequently excised by the otolaryngologist [9]. The presence of multiple specialties within the operating theatre complex is a boon under such circumstances. The timely intervention by the otolaryngologist, as in our case, allowed the surgery to proceed as planned. Furthermore, any future exposure to anaesthesia warrants special precautions, including a thorough clinical assessment for signs of respiratory distress, microlaryngoscopy, and computed tomography imaging to assess for any residual subglottic web. The parents were informed of these necessary precautions.

Tracheal webs can also be acquired as a rare sequela of prolonged invasive ventilation. Ajimi J et al. encountered a case of acquired subglottic laryngeal web that led to failed intubation, after which the patient was allowed to awaken. Laryngeal microsurgery was successfully performed the next day, and cholecystectomy was carried out a few days later [10]. The presence of post-resection glottic edema should also be thoroughly assessed, as it may lead to difficult extubation or postoperative respiratory distress.

In elective surgical settings, particularly in resource-constrained environments, it is ideal to abandon the procedure upon encountering a laryngeal web, followed by a thorough airway evaluation and a planned, controlled excision of the web at a later stage. However, in emergency surgeries where the airway must be secured promptly, the use of a SGAD to maintain ventilation, followed by careful excision of the web, may allow the procedure to continue as intended. If ventilation cannot be maintained through non-invasive methods, emergency invasive front-of-neck access becomes mandatory to secure the airway and prevent hypoxia.

The current case highlights the intricacies of managing an unanticipated difficult airway due to a congenital laryngeal web in pediatric patients. It also emphasizes the importance of avoiding repeated intubation attempts upon encountering resistance in the subglottic region. The case was successfully managed with the use of fiberoptic bronchoscopy and the collaborative efforts of a multidisciplinary team.

## Conclusions

The laryngeal web is a rare yet challenging laryngeal malformation. It must be recognized and investigated in infants who present with recurrent stridor or prolonged hoarseness after birth, or later in life, depending on the grade of the laryngeal web. The treatment plan, such as endoscopic web removal or surgical excision, should be selected based on the classification of the laryngeal web. This case highlights the importance of prompt preparation for unanticipated airway management and the need for coordinated teamwork with other departments, such as the otolaryngology surgical team, to save patients’ lives.

## References

[1] Avelino MA, Pazinatto DB, Rodrigues SO, Maunsell R (2022). Congenital laryngeal webs: from diagnosis to surgical outcomes. Braz J Otorhinolaryngol.

[2] Lawlor CM, Dombrowski ND, Nuss RC, Rahbar R, Choi SS (2020). Laryngeal web in the pediatric population: evaluation and management. Otolaryngol Head Neck Surg.

[3] McElhinney DB, Jacobs I, McDonald-McGinn DM, Zackai EH, Goldmuntz E (2002). Chromosomal and cardiovascular anomalies associated with congenital laryngeal web. Int J Pediatr Otorhinolaryngol.

[4] Yamamoto S, Tetsuka K, Sato Y, Endo S (2010). Unsuspected tracheal web inhibits endotracheal intubation: report of a case. J Anesth.

[5] Jefferson ND, Cohen AP, Rutter MJ (2016). Subglottic stenosis. Semin Pediatr Surg.

[6] Milczuk HA, Smith JD, Everts EC (2000). Congenital laryngeal webs: surgical management and clinical embryology. Int J Pediatr Otorhinolaryngol.

[7] Chong ZK, Jawan B, Poon YY, Lee JH (1997). Unsuspected difficult intubation caused by a laryngeal web. Br J Anaesth.

[8] Stein ML, Park RS, Kovatsis PG (2020). Emerging trends, techniques, and equipment for airway management in pediatric patients. Paediatr Anaesth.

[9] Nguyen NK (2006). Unexpected tracheal web encountered during difficult intubation in the operating room. Proc (Bayl Univ Med Cent).

[10] Ajimi J, Nishiyama J, Ando A, Suzuki T (2013). Unexpected difficult intubation owing to a tracheal web in a patient with past history of a Fontan procedure. J Anesth.

